# Automated Optical Treatment Based on Diffuse Reflectance Signals

**DOI:** 10.1002/jbio.70363

**Published:** 2026-09-14

**Authors:** Callum D. Appel, Mark S. Carlsen, Laura L. Lukov, Chi Zhang

**Affiliations:** ^1^ James Tarpo Jr. and Margaret Tarpo Department of Chemistry Purdue University West Lafayette Indiana USA; ^2^ Purdue Institute for Cancer Research West Lafayette Indiana USA; ^3^ Purdue Institute of Inflammation, Immunology, and Infectious Disease West Lafayette Indiana USA

**Keywords:** bacterial inhibition, closed‐loop, diffuse reflectance, lasers, optical treatment, pigment removal

## Abstract

Laser‐based therapies offer high spatial precision and minimal invasiveness but rely heavily on operator skill and continuous manual guidance. Inaccurate operation can lead to undesired laser damage to surrounding healthy tissue. Closed‐loop feedback approaches have been explored in laser ablation but remain underdeveloped for applications such as pigment removal and bacteria inactivation. Here, we introduce signal‐activated precision optical treatment (SPOT), a high‐speed closed‐loop feedback platform based on diffuse reflectance detection. A dedicated and modulated guidance laser continuously interrogates tissue reflectance, and lock‐in detection selectively extracts the guidance laser reflectance while effectively rejecting ambient light interference. This design enables high sensitivity to small contrast differences and sub‐microsecond response. We demonstrate automated pigment removal using a nanosecond laser and bacterial inhibition with a blue laser. The SPOT framework is adaptable to other optical signal detection, enabling automated, safer, and more precise laser therapies that minimize damage to healthy tissue.

## Graphical abstract

An automated laser treatment system guided by diffuse reflectance signals is introduced. The closed‐loop platform suppresses ambient light interference through modulated illumination and lock‐in detection, enabling high sensitivity, microsecond response time, and automated laser activation. This approach improves treatment precision and safety for applications including pigment removal and bacterial inactivation.

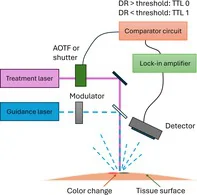

## 1 Introduction

Laser‐based treatments are widely employed across modern medical disciplines, including dermatology, dentistry, oncology, and surgery, owing to their high spatial precision and minimal invasiveness [1, 2, 3, 4, 5, 6, 7]. The first major category of laser treatments involves high‐energy laser irradiation that induces irreversible tissue or material changes through extreme photothermal, photo‐plasmonic, and photomechanical interactions. Representative examples include laser ablation, tattoo removal, and laser lithotripsy, where the therapeutic outcome is achieved by tissue vaporization, fragmentation, or disruption [8, 9]. The second major category operates at relatively low laser energy densities, usually below the threshold of cellular damage, and is generally referred to as photobiomodulation (PBM) [10, 11, 12]. The underlying mechanisms include mild photothermal and/or photochemical effects that trigger cellular signaling pathways and other non‐destructive biological responses. This approach has demonstrated efficacy in soft‐tissue healing, tissue regeneration, and inflammation reduction [12]. In clinical practice, PBM is commonly implemented as low‐level laser therapy (LLLT) [13, 14] or high‐intensity laser therapy (HILT) for rehabilitation purposes [15, 16]. The third major category is photodynamic therapy (PDT), which requires the combined action of light, molecular oxygen, and photosensitizers [17, 18, 19]. PDT produces therapeutic effects through light‐activated photochemical reactions, primarily via reactive oxygen species (ROS) generation, and is widely applied in dermatological treatments [20, 21, 22, 23]. The fourth category, which differs from PDT and does not rely on exogenous photosensitizers but instead on intrinsic light‐absorbing chromophores, is the use of short‐wavelength light for bacterial inhibition [24, 25, 26]. The antimicrobial effects of blue light are mediated through light absorption by endogenous biomolecules in bacteria, leading to ROS generation and effectively reducing bacterial burden to promote tissue healing and recovery.

For many laser‐based therapies, particularly high‐energy laser radiation, bacterial inhibition, and certain PDT applications, precise spatial confinement of light delivery is essential, as inadvertent irradiation can result in undesired damage to surrounding healthy tissue [27]. However, in most existing instruments and clinical workflows, treatment accuracy, safety, and reproducibility remain heavily dependent on operator experience and manual control. Conventional systems typically rely on predefined parameters and continuous operator guidance, limiting their ability to adapt to dynamic changes in tissue properties or target motion during treatment. These limitations increase the risk of overtreatment and unintended collateral damage.

To improve treatment precision and safety, closed‐loop feedback control has been explored primarily in the context of laser ablation surgery [28, 29, 30, 31, 32]. Most prior efforts have focused on monitoring optical signatures generated during laser ablation to distinguish tissue types and automatically terminate irradiation upon the detection of unwanted tissue. For example, Kim et al. reported an optical feedback system based on tissue fluorescence and plasma luminescence to guide ultrashort‐pulse laser ablation, enabling discrimination between bone and spinal cord tissue [28]. Nahen and Vogel demonstrated that acoustic signals generated during ablation can serve as real‐time feedback for skin ablation [29]. Rupprecht et al. employed shockwave and optical emission signals to guide Nd:YAG laser ablation [30]. Scattering‐based approaches such as diffuse reflectance spectroscopy (DRS) have also been applied to tissue differentiation and ablation guidance [31, 32], while optical coherence tomography (OCT) has recently been explored for tissue‐type discrimination during laser ablation [33, 34]. In addition, temperature monitoring during ablation is critical for preventing overheating, and closed‐loop thermal feedback has been demonstrated using optical and photoacoustic approaches [35, 36]. Korganbayev et al. demonstrated the feasibility of closed‐loop laser ablation using high‐density fiber Bragg grating arrays for real‐time temperature feedback and spatial temperature regulation for laser ablation of hepatic tissue [37]. They later advanced this framework by integrating a numerically optimized proportional‐integral‐derivative (PID) controller, enabling more accurate regulation of tissue temperature across different treatment locations [38]. Beldek et al. developed a closed‐loop laser ablation system that regulates laser intensity based on intensity distribution measurement [39]. Beyond laser ablation, multi‐wavelength photoacoustic temperature feedback was developed to enable real‐time temperature measurement and closed‐loop control of photothermal therapy, achieving sub‐degree temperature accuracy and improving treatment precision while reducing thermal damage to surrounding tissue [40]. Phan et al. reported the development of a real‐time closed‐loop photothermal therapy system integrating infrared thermal sensing, artificial neural network prediction, and fuzzy logic control enabled precise temperature regulation and accurate induction of predefined thermal damage while minimizing injury to surrounding tissue [41]. Furthermore, preliminary work also demonstrated optical feedback‐controlled infrared laser delivery for vascular healing [42].

Despite these advances, feedback‐controlled laser treatment remains largely unexplored for pigment removal and bacterial inhibition. In such applications, treatments are typically confined to tissue surfaces, making diffuse reflectance (DR) a particularly attractive modality [43]. However, DR measurements in clinical environments are often significantly affected by ambient room lighting, reducing sensitivity and limiting the ability to detect subtle spectral differences between diseased and healthy tissue. Furthermore, many existing feedback strategies rely on optical or acoustic signals generated by the treatment laser itself, meaning that some degree of unintended damage must occur before corrective action can be taken.

Here, we report signal‐activated precision optical treatment (SPOT), a high‐speed closed‐loop optical treatment system that leverages opto‐electronic feedback to achieve fast response and high sensitivity. A dedicated guidance laser provides real‐time interrogation of tissue reflectance properties and delivers feedback on a microsecond timescale, thereby minimizing overtreatment. By incorporating modulation and lock‐in detection, the system is rendered insensitive to ambient lighting and therefore gives high sensitivity to small reflectance differences associated with pigments or chemical composition. Using model systems, we demonstrate automated pigment removal with a nanosecond Nd:YAG laser and bacterial inhibition using 405‐nm laser. The optical signals used for target discrimination in SPOT can be potentially extended beyond DR, providing other contrasts reflecting unique tissue physical or chemical signatures. SPOT enables automated, spatially precise, and selective treatment with minimal collateral damage, offering a generalizable framework for automated and safer laser therapies in pigment removal, antimicrobial light treatment, PDT, and beyond.

## 2 Methods

The SPOT technology developed here integrates a closed‐loop feedback system that uses optical signals from sample surfaces (primarily DR reported here) to determine the activation of the treatment laser. Two treatment modalities are applied to demonstrate the concept: pigment removal and bacterial inactivation.

**FIGURE 1:**
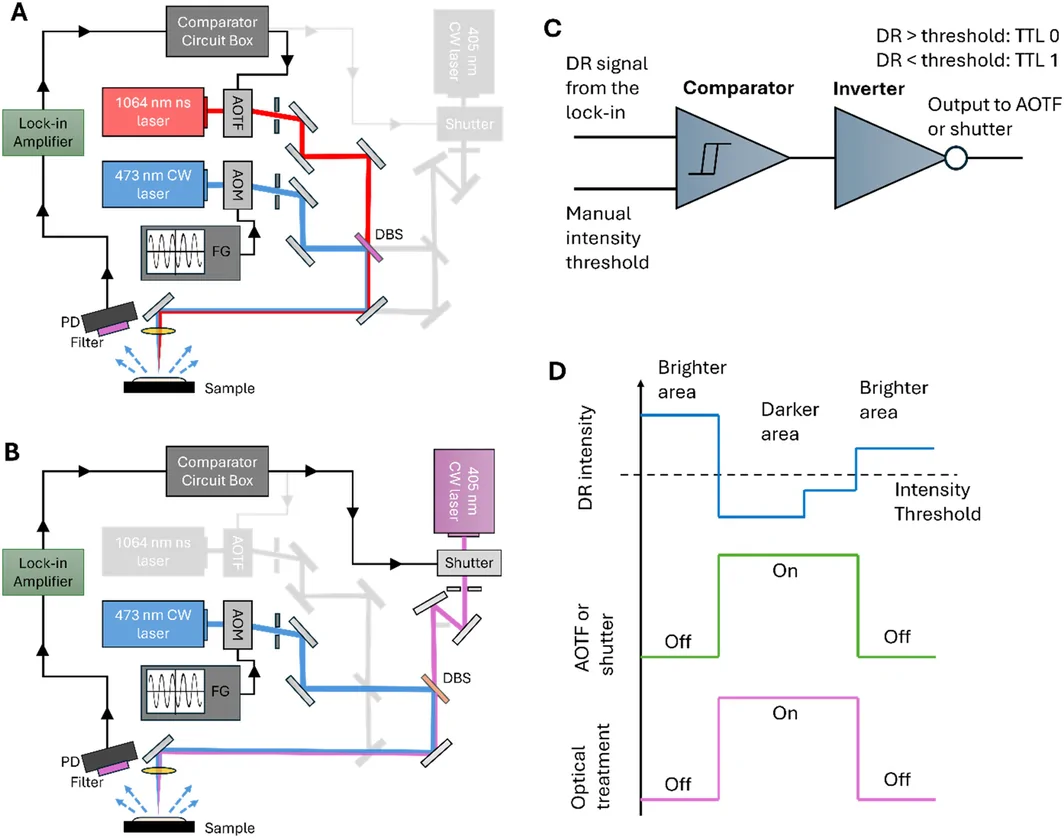
Schematic of the signal‐activated precision optical treatment (SPOT) system. (A) Optical configuration of the SPOT system for pigment ablation, using a nanosecond pulsed laser as the treatment laser. AOTF, acousto‐optic tunable filter; AOM, acousto‐optic modulator; CW, continuous wave; DBS, dichroic beam splitter; FG, function generator; PD, photodiode. (B) Optical configuration of the SPOT system for bacterial inhibition, using a 405 nm continuous‐wave laser as the treatment laser. (C) Workflow of the SPOT command processing, in which the detected diffuse reflectance (DR) signal is processed by a comparator circuit and an inverter to control the treatment lasers. (D) An illustration of the DR signal, preset intensity threshold, AOTF or shutter command, and the resulting optical treatment at brighter and darker regions of the sample. When the DR intensity exceeds the threshold, the gating component remains off, and no treatment is applied; when the DR intensity falls below the threshold, optical treatment is triggered and delivered to the sample.

The optoelectronic configuration of the SPOT system for pigment removal is shown in Figure 1A. A 473 nm guidance laser (MBL‐FN‐473‐100 mW, CNI lasers) scans the sample surface and generates DR signals that vary according to the tissue surface reflectance. The lasers were directed to the surface with a 0° incidence angle. The laser beam was focused to about 1.00 mm in diameter using a lens with a 100 mm focal length. The spot size can be adjusted by varying the position of the focusing lens with a manual translation stage or changing the beam width before the lens. The laser power delivered to the sample was 3.5 mW. This is the maximum laser power our system can deliver for the 473 nm guidance laser using IR optics optimized for the IR treatment laser. At this power level, the phototoxic effects on biological tissue samples are minimal. The DR signals of the 473 nm laser are detected by a large‐area (10 × 10 mm^2^) photodiode (PD, S3994 Hamamatsu) with a 1064 nm notch filter, which collects both the guidance laser DR and ambient light. The PD was facing the laser/sample interaction spot and positioned 60 mm from the spot at a 45° reflection angle relative to the incident lasers. This configuration was chosen to maximize the DR signal while maintaining a practical working distance. A closer PD distance to the sample could improve the DR signal intensity and signal‐to‐noise ratio (SNR). The PD position and angle can be tuned by adjusting the mechanical mounts.

To suppress interference from ambient lighting, the guidance laser is intensity‐modulated at a high frequency (1.22 MHz) using an acousto‐optic modulator (AOM, M1205‐P80L‐1 Isomet). The modulated DR signal corresponding specifically to the guidance laser is then extracted from the PD output using a digital lock‐in amplifier (MFLI 5 MHz, Zurich Instruments). This high‐frequency modulation effectively removes the ambient light background and operates within a frequency range where laser intensity noise is minimal. The lock‐in amplifier output is fed into a comparator circuit box (Figure S1), where the DR signal is compared with a preset threshold [44]. The “invert” function in the comparator circuit box enables DR signals below the threshold (corresponding to target pigment regions) to generate a TTL high output. The threshold is determined by measuring the DR signal voltage of the target of interest versus the surrounding area and tissue. When the target and background have a larger DR signal difference (over 20% darkness), the threshold is set to be 10 mV above the target signals. This TTL command is sent to an acousto‐optic tunable filter (AOTF, PCAOM NIR 1 G&H) to activate the 1064 nm Nd:YAG nanosecond laser (Polaris II, New Wave Research), which is spatially combined with the guidance laser using a dichroic beam splitter. The nanosecond treatment laser delivered about 2.10 mJ/pulse to the sample at a rate of 20 pulses per second. The feedback response time from signal detection to treatment delivery is within microseconds, mainly determined by the lock‐in amplifier time constant. This fast response time ensures that treatment is delivered precisely at the target locations identified by the guidance laser. The samples were scanned using two orthogonally mounted motorized linear stages (X‐LSM050A, Zaber Technologies, and MTS50‐Z8 Thorlabs). Stage motion and raster scanning were controlled using custom‐written code.

In addition to the 1064 nm nanosecond treatment laser source, a 405 nm continuous‐wave (CW) laser (MDL‐III‐405‐100 mW CNI lasers) can be selected as the treatment beam for selective bacteria inhibition (Figure 1B). The 405 nm laser follows a similar optical path and activation mechanism as the nanosecond laser, enabling localized treatment of bacteria associated with tissue DR difference (such as redness during infection or inflammation). Due to the longer treatment time requirements for bacteria inactivation, a mechanical shutter was applied to control the activation/termination of the 405 nm treatment laser. The response time of the mechanical shutter is approximately 10 ms.

Figure 1C illustrates the workflow by which the DR signal extracted by the lock‐in amplifier commands the AOTF or shutter to control activation of the treatment laser. Figure 1D presents an illustration showing how regions with different DR levels (brighter or darker areas) generate corresponding command signals that control the treatment laser delivery on the sample. In the current implementation, the system is configured to activate the treatment laser at darker spots. However, this logic can be reversed by switching the inverter, enabling selective treatment of brighter regions within a darker background or within a contrast range. The spatial precision of the SPOT system is primarily determined by the focal spot sizes of the guidance and treatment laser beams, which were both focused to approximately 1 mm in this work. The spatial precision can be adjusted by changing the focal spot size through translation of the lens‐mounting stage. The optimal spatial precision should be selected according to the application, balancing the target size and treatment speed. Furthermore, the effective precision is also influenced by the spatial overlap quality of the guidance and treatment beams. A poor overlap will deteriorate precision.

## 3 Results

### 3.1 System Evaluation

**FIGURE 2:**
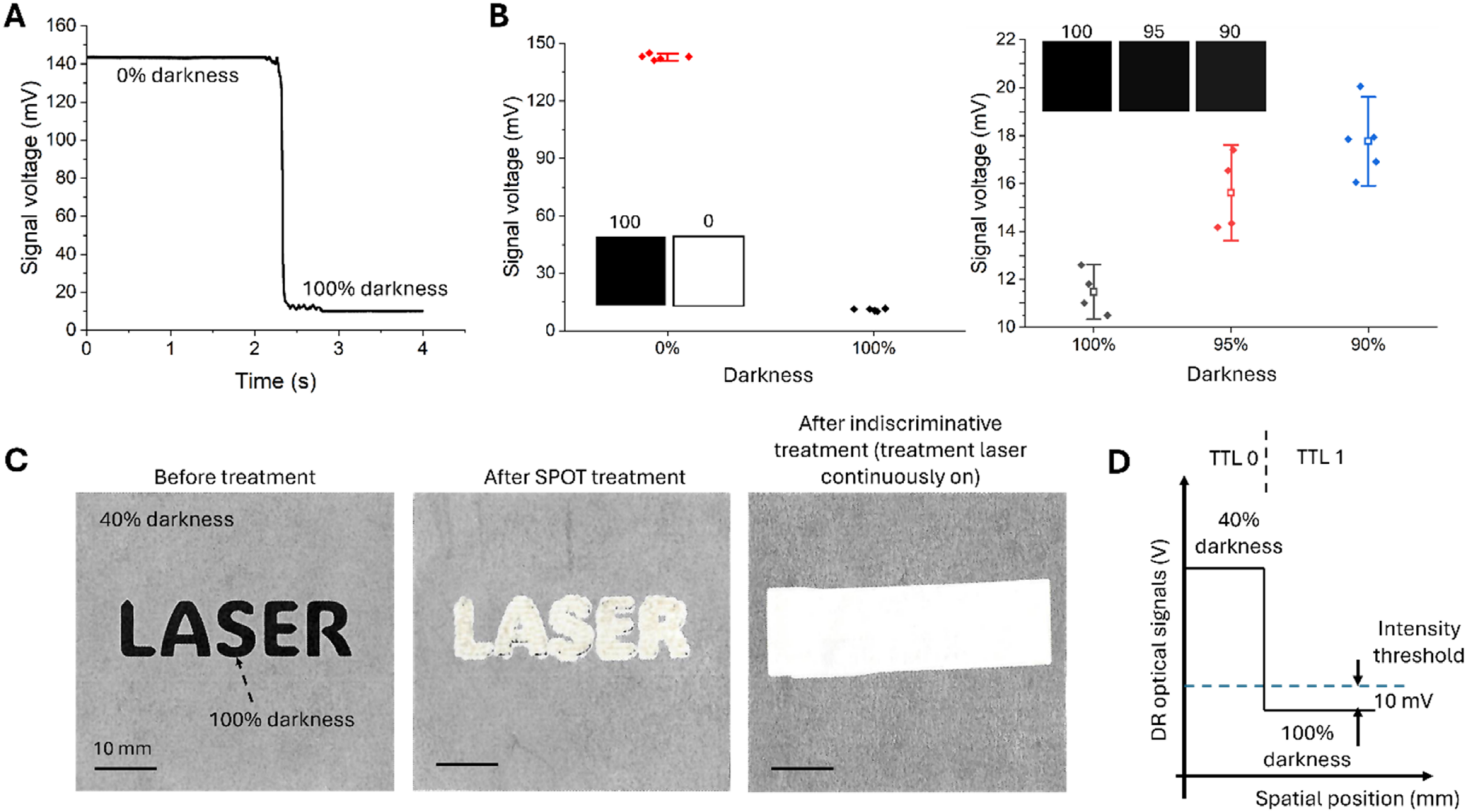
Evaluating the sensitivity and automation of the SPOT system. (A) Detected DR optical response after lock‐in extraction when the guidance laser is moved from plain paper (145 mV, 0% darkness) to a black ink printed area (11.3 mV, 100% darkness). (B) Quantitative signal voltages corresponding to 100% and 0% (left), and 100%, 95%, and 90% (right) darkness from printed squares. Dots are measurement values; bars are 1.5 interquartile range. (C) Letters printed with 100% darkness on a gray background (40% darkness) using a laser‐jet printer on white paper for testing system automation (left). The printed sample after SPOT treatment with a threshold set between the letters and the background, so that only the letters are treated (middle). The sample after treatment with the nanosecond treatment laser continuously on during sample scanning (right). (D) An illustration of the intensity threshold selected for pigment removal in Panel C.

We first tested the performance of the SPOT system using black ink printed on paper using a laser‐jet printer. The darkness scale is shown in Figure S2. The SPOT system can achieve an SNR of approximately 193 for black ink with 100% darkness (Figure 2A). Considering SNR = 3 as the limit of detection, the lowest resolving darkness difference is around 100%/(193/3)≈1.5%. It is worth noting that DR signals measured from the same grayscale square exhibit fluctuations due to variations in paper scattering and printing nonuniformity across different spatial positions. The left panel of Figure 2B presents quantitative comparisons of 100% and 0% darkness measured at multiple locations, showing slight variations in signal levels. The right panel of Figure 2B compares DR signal intensities for 100%, 95%, and 90% darkness. Although the theoretical resolving power is 1.5% darkness, these intensity fluctuations limit the practical differentiation capability for printed paper patterns. Variations in printing quality and paper reflectance inhomogeneity allow for reliable discrimination only at approximately 5%–10% differences in darkness without signal overlap. For distinguishing a 10% difference in darkness, an intensity threshold of ~2 mV above the target signal was applied.

To demonstrate automated treatment laser responses based on DR signals, we used laser‐printed letters on white paper (Figure 2C) as a model system and showed that a nanosecond laser in the SPOT system can automatically remove targets with desired darkness (Video S1). The background was printed at a grayscale level of 40% darkness, while the letters were printed at 100% darkness. A voltage threshold was defined using the comparator circuit to distinguish between the two grayscale levels. The threshold was set at 10 mV above the DR signal voltage from the letter target (Figure 2D). In practice, threshold values within a range spanning approximately 10 mV above the target DR signal and 10 mV below the DR signal from the non‐target regions can be used. Under this condition, SPOT enabled fully automated and unsupervised removal of the darker letters without affecting the lighter background (Figure 2C middle panel, Video S1). In contrast, when the SPOT function was disabled, the nanosecond laser indiscriminately ablated all pigments within the rectangular scan area (Figure 2C right panel). This proof‐of‐concept experiment demonstrates that SPOT minimizes overtreatment by restricting laser irradiation to regions selected by the DR signal and intensity threshold defined in the comparator circuit. Such spatially selective activation has the potential to reduce collateral damage to surrounding healthy tissue compared with conventional non‐selective laser treatment approaches.

### 3.2 Selective Pigment Removal

**FIGURE 3:**
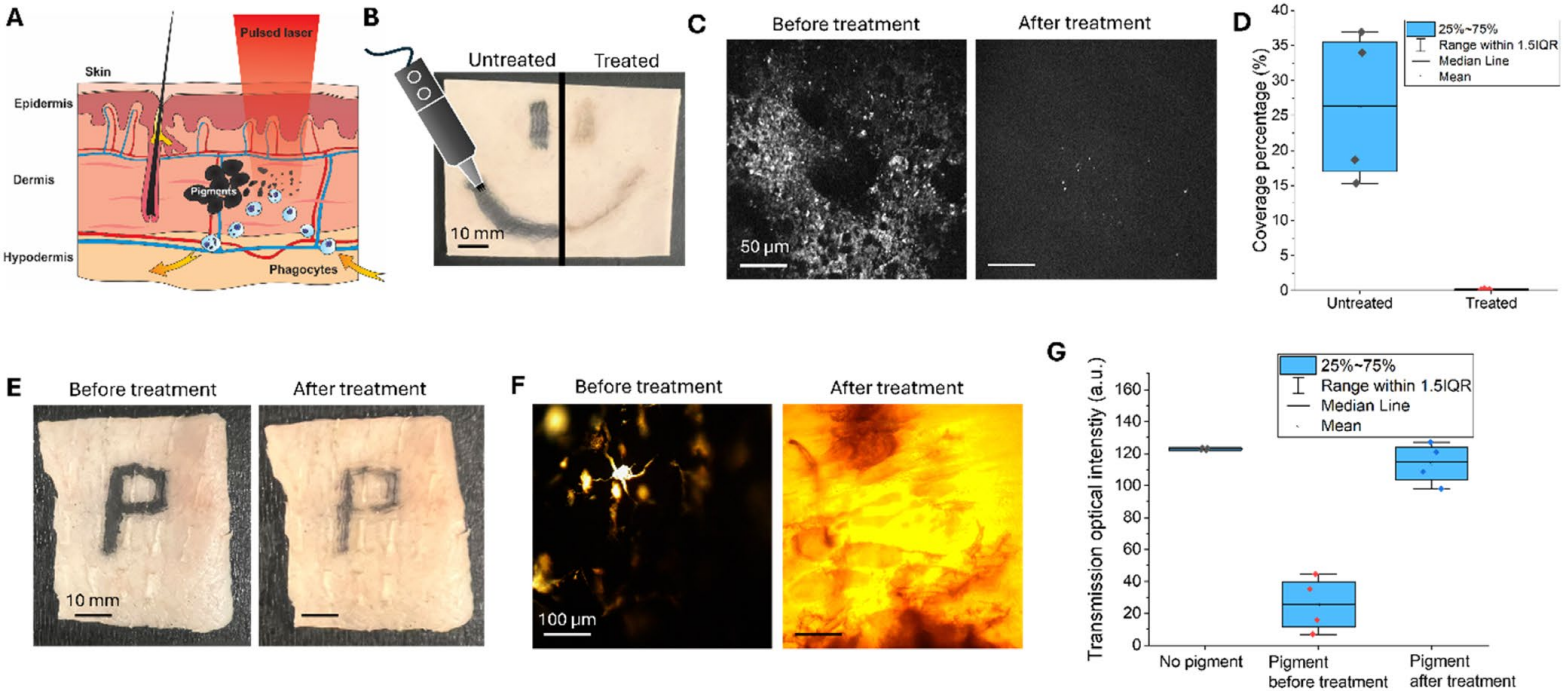
Pigment breakdown and removal using SPOT. (A) Schematic of the mechanism of pigment breakdown and removal in skin. Pulsed laser irradiation fragments pigment aggregates into smaller particles that can be removed by phagocytes for clearance. (B) Black pigments are injected into synthetic practice skin using a tattoo gun before and after nanosecond laser treatment. (C) Epi‐TA images of the black pigments from the untreated and treated areas. (D) Quantitative analysis of pigment removal by SPOT for inks in synthetic practice skin based on epi‐Transient absorption (TA) signals. Dots are measurement results; bar graphs plot the 25%–75% value range. IQR, interquartile range. (E) Black pigments injected into porcine skin using a tattoo gun before and after nanosecond laser treatment. (F) Bright‐field transmission images of pigment‐injected areas before and after nanosecond laser treatment in Panel E. (G) Quantitative analysis of pigment removal by SPOT for inks in procine skin based on bright‐field transmission images. Dots are measurement results; bar graphs plot the 25%–75% value range. IQR, interquartile range.

Skin pigment removal is a multistep process and is typically achieved using either picosecond or nanosecond laser sources [45, 46, 47]. The treatment laser penetrates through the epidermis of the skin to the dermis, where the pigments accumulate. Nanosecond lasers use both photothermal and photomechanical processes initiated by pulsed laser absorption to fragment pigments into smaller particles. This process induces controlled cellular disruption, which facilitates the recruitment of the immune system to clear the fragmented pigments over time. In contrast, picosecond lasers achieve pigment fragmentation primarily through photomechanical processes. These mechanisms are illustrated in Figure 3A. In this study, a 1064 nm Q‐switched nanosecond laser was used for pigment removal.

The nanosecond treatment beam delivered a maximum pulse energy of ~2.1 mJ to the sample. By adjusting the focus, the optical intensity was set to ~270 mJ/cm^2^ with a beam diameter of 1 mm. Although this laser dosage is lower than that used in commercial laser pigment removal systems (typically 1–10 J/cm^2^) [48], it is sufficient for pigment removal in our study. SPOT was used to automatically perform treatment based on pigment deposition. The treatment laser was triggered by pigment detection using a threshold set at 10 mV above the DR signal from the pigmented regions.

To assess pigment fragmentation and clearance induced by the nanosecond laser, we used transient absorption (TA) microscopy to image the pigment depositions [48]. Details of the TA instrumentation used in this work are provided in Figure S3. Pigments exhibit strong TA signals due to their broadband absorption. To avoid strong stimulated Raman scattering signals from the sample, an optical delay of ~30 ps was introduced between the pump and probe pulses.

To mimic pigment removal in skin tissue, we first used a tattoo gun to inject black pigments into a synthetic tattoo practice skin at a depth of 0.5 mm and treated half of the pattern using SPOT. A pronounced decrease in ink visibility was observed on the treated half of the sample (Figure 3B). Because of the substantial thickness of the synthetic skin, TA signals were collected in the epi‐direction (Figure S3). In the TA microscopy images, only small and fragmented particles remained on the treated side (Figure 3C). Quantitative analysis of the epi‐TA signals is shown in Figure 3D, clearly demonstrating the efficiency of pigment removal.

To further evaluate the treatment under more realistic conditions, we performed the same black pigment injection in porcine skin using the tattoo gun, followed by SPOT treatment. Similarly, SPOT resulted in a drastic reduction in pigment darkness after treatment (Figure 3E). Transmission brightfield images are also a good metric to evaluate pigment removal efficacy. As shown in Figure 3F, example transmission brightfield images from the tissue sample of the pigmented area before and after treatment clearly show the pigment removal by the nanosecond laser. Quantitative comparison of transmission intensity across different regions shows a significant recovery of optical transparency after treatment (Figure 3G), resulting from the breakdown and removal of pigments in the tissue.

### 3.3 Selective Bacterial Inhibition

**FIGURE 4:**
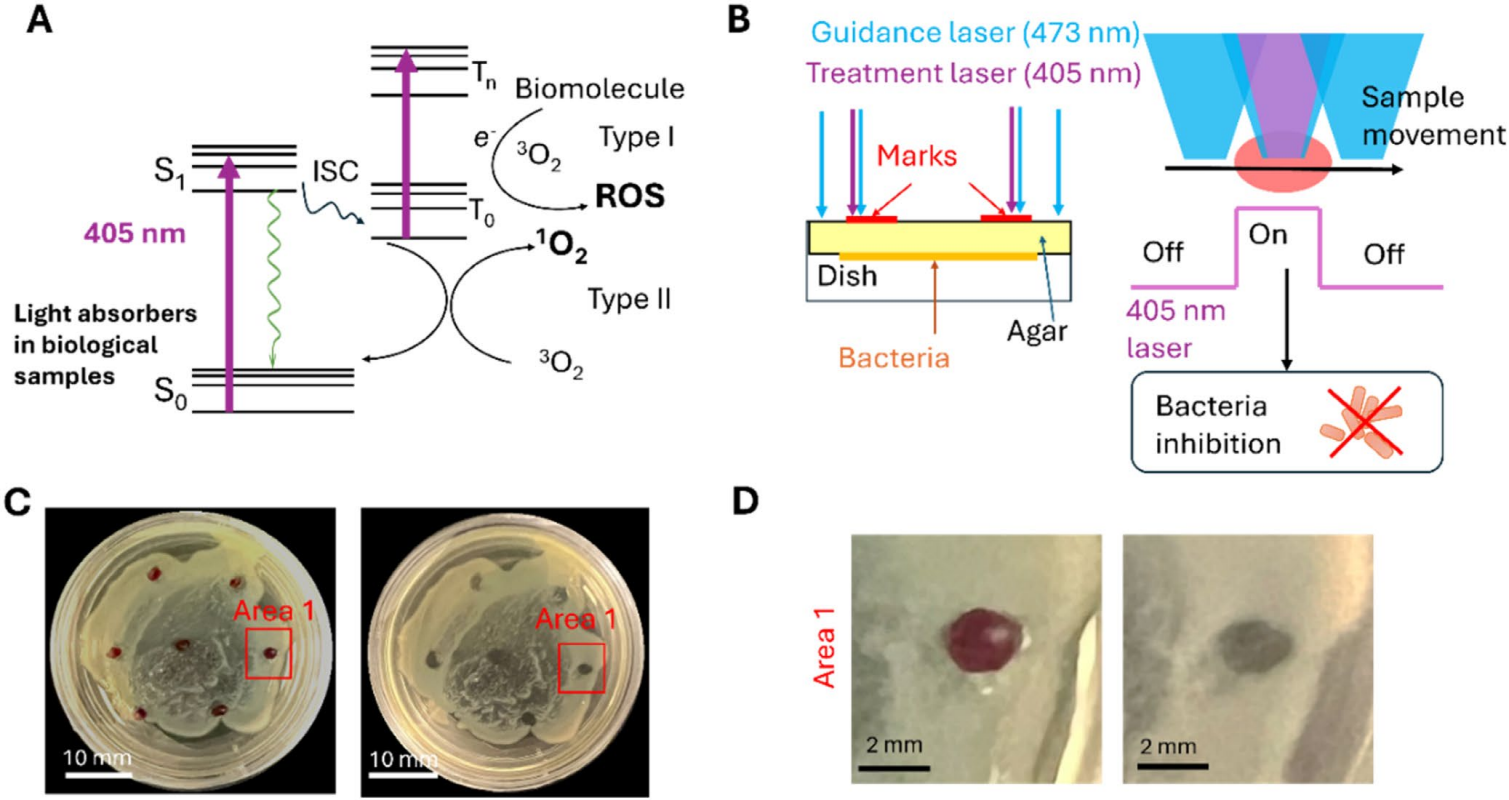
Inhibition of *Pseudomonas aeruginosa* using SPOT. (A) Jablonski diagram illustrating 405 nm laser absorption by biomolecules in the bacteria, followed by intersystem crossing (ISC) to the triplet state and the subsequent generation of reactive oxygen species (ROS) and singlet oxygen. (B) Illustration of targeted bacterial inhibition guided by red marks added to the bottom of the bacterial culture Petri dish. (C) Photographs of the Petri dish culturing *Pseudomonas aeruginosa* after SPOT treatment, before (left) and after (right) the removal of the red marks. (D) Magnified views of the selected regions in Panel C.

To further demonstrate the capability of SPOT, we applied it to achieve spatially selective bacterial inhibition using a 405 nm blue CW laser. Human skin serves as the first barrier against pathogens, and bacterial infections of the skin are among the most encountered skin diseases that require timely and active treatment. Such infections often cause visible color changes, such as red spots or patches, as observed in diseases such as impetigo, folliculitis, and erysipelas. Blue light has been demonstrated to effectively inhibit bacterial growth and kill bacteria [24, 25]. The antimicrobial effect is primarily associated with the generation of ROS and singlet oxygen [49, 50]. Many bacteria naturally produce light‐absorbing molecules such as porphyrins, flavins, and other pigments that strongly absorb blue light. Upon excitation, these molecules can undergo intersystem crossing to form a long‐lived triplet excited state, facilitating the production of singlet oxygen and other ROS, which subsequently damage essential biomolecules including cell membranes, proteins, and DNA. The mechanisms of bacterial inhibition and collateral mammalian cell damage are illustrated in Figure 4A.

Despite its effectiveness, blue light (e.g., 405 nm) can also generate ROS in mammalian tissue. Endogenous chromophores such as flavin and cytochrome c in mitochondria can absorb blue light [51] and induce ROS generation, potentially damaging mitochondria and other cellular structures [52]. Therefore, collateral tissue damage may occur if the light is not delivered selectively to the intended locations. To improve treatment efficacy while minimizing damage to surrounding tissue, SPOT can utilize bacteria‐induced skin color changes to selectively activate treatment only at infected regions.

*Pseudomonas aeruginosa* ( *P. aeruginosa* ) was used in this study. There are many strong blue‐light absorbers in *P. aeruginosa*, such as flavin, pyoverdine, and pyocyanin. Blue light irradiation can induce phototoxicity through the strong absorption of these molecules. The bacteria were first homogeneously spread on the cured agar (~0.8 mm thickness) surface in a 35 mm Petri dish. Red spots were then marked on the bottom of the dish to mimic the color changes observed in infected skin regions and to ensure no perturbation to the bacterial culture. The red marks used here, despite being preliminary, can represent a color change effect induced by various skin infections such as impetigo, erythrasma, or cellulitis. The sample was translated across the overlapped laser spots using a two‐dimensional scanning stage, and the 405 nm treatment laser (75 mW) was activated only when the guidance laser (473 nm) detected the red‐marked areas. During treatment, the scanning was paused to provide a total irradiation time of 120 s at the target locations to ensure effective bacterial inhibition. A schematic illustration of the experimental setup is shown in Figure 4B. Following treatment, the bacteria were allowed to grow overnight to form colonies, enabling a comparison of the inhibition effects between treated and untreated areas.

Figure 4C shows photographs of the bacterial culture Petri dish with red marks (left) added to the bottom of the dish after SPOT, and after the marks were removed (right). The inhibition process can be observed from the reflection contrast between the treated areas and the surrounding untreated regions (Figure 4C). Magnified photographs of a selected region from Figure 4C are shown in Figure 4D for clearer visualization.

**FIGURE 5:**
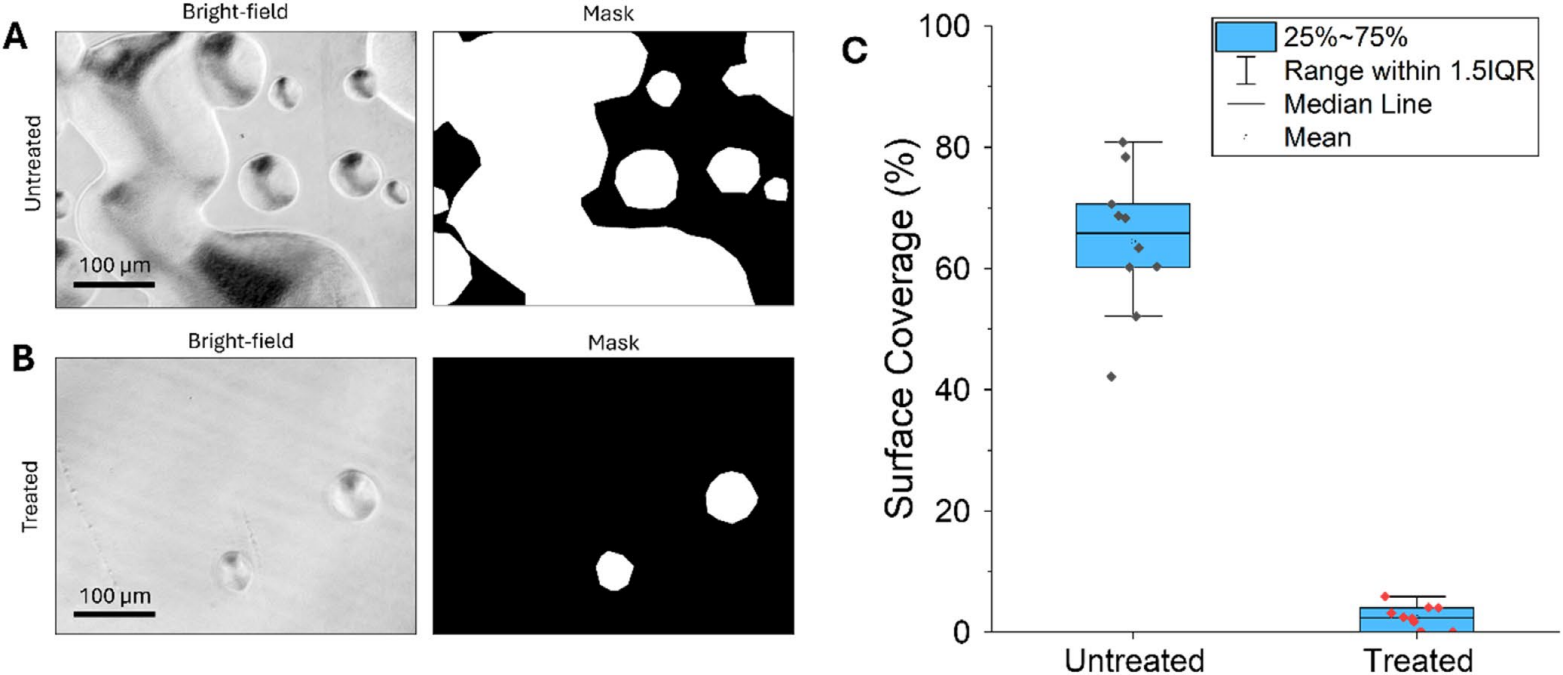
Quantitative analysis of *Pseudomonas aeruginosa* inhibition by the 405 nm CW laser. (A) Bright‐field transmission microscopy image and the corresponding colony segmentation mask (white color) from a representative field of view (FOV) of bacterial colonies grown in an untreated region of the agar plate. (B) Bright‐field transmission microscopy image and the corresponding segmentation mask from a representative FOV of colonies grown in a region treated with the 405 nm laser. (C) Quantitative comparison of bacterial colony surface coverage measured from eight FOVs in the treated and untreated groups. Dots are measurement results; bar graphs plot the 25%–75% value range. IQR, interquartile range.

We then used transmission microscopy to compare images from the treated and untreated areas. Untreated regions exhibited dense and continuous coverage of bacterial colonies (Figure 5A). In contrast, such colonies were mostly absent in the SPOT‐treated regions (Figure 5B). The bacterial inhibition efficacy was analyzed by comparing surface coverage of the colonies from the treated and untreated locations. The quantitative analysis of the surface coverage (Figure 5C) was performed by selecting colony masks (Figure 5A,B) based on transmission optical imaging of *P. aeruginosa* colonies grown on the agar plate. Eight FOVs in the untreated and treated cases used for the analysis are detailed in Figures S4 and S5.

## 4 Discussion

The results presented here demonstrate the ability of SPOT to achieve automated and spatially selective treatment. SPOT is highly adaptable to multiple laser‐based treatments and provides a high response time of microseconds. The use of lock‐in detection provides superior sensitivity, and the use of a guidance beam to determine activation minimizes unwanted tissue damage. We demonstrate the capabilities of SPOT through applications in pigment removal and bacterial inactivation. Unlike existing systems that rely on damage‐induced signals from non‐target tissue to terminate the treatment laser [28, 29], SPOT enables preemptive control, thereby reducing unintended damage to surrounding healthy areas.

For the detected DR signal, a portion of the guidance laser is reflected directly from the skin surface, while another portion penetrates the tissue and undergoes multiple scattering events within the epidermal and dermal layers before re‐emerging. As a result, DR can probe targets located up to a few millimeters under the skin or tissue surface. This enables the detection of features such as pigment accumulation and inflammation‐induced redness within these layers. In contrast, the effective penetration depth of the treatment laser depends on its wavelength and power. In general, IR lasers achieve greater penetration depths than visible or UV wavelengths.

For colored targets, however, the sensitivity of SPOT depends strongly on the spectral reflectance of the material. If a target color efficiently reflects 473 nm light (has blue color), the resulting contrast will be reduced, leading to lower detection sensitivity with this guidance wavelength. In such cases, selecting a guidance laser with a wavelength that is more strongly absorbed (or less reflected) by the target would improve contrast and enhance detection performance.

The sensitivity achieved for ink printed on paper may not be directly translated to practical conditions for skin pigment removal. In the model system, the paper surface is flat and has a more consistent roughness, and both the angle and distance between the surface and the photodiode remain largely constant. In practical dermatological treatment conditions, tissue may present varying surface roughness, and small variations in surface position and angle relative to the photodiode are also likely to occur. As a result, the effective differentiation capability of SPOT in practical dermatological applications may be different from the model system.

Regarding pigment removal, we only demonstrated treatment in model systems and sectioned dead skin tissues. Such in vitro demonstrations do not fully represent the efficacy of in vivo skin treatment using pulsed lasers. In clinical laser‐based pigment removal, fragmented pigments are gradually cleared by white blood cells. These in vivo effects can only be observed in living animals or humans with active blood flow and immune responses. Future work will extend this concept to in vivo systems and evaluate its integration with modified commercial laser treatment equipment.

There is still room for further improvement of the SPOT technology. First, the current implementation relies on moving the sample while the laser beam remains stationary. It has not yet incorporated laser beam scanning to improve treatment speed. The low repetition rate of the nanosecond laser used in this work is not optimally compatible with high‐speed beam scanning. Nevertheless, beam scanning is currently a standard approach in nanosecond laser treatments. SPOT integration with the beam scanning apparatus will be explored in future developments. Furthermore, tissue scattering properties, laser incidence angle, and photodiode position may affect the DR detection efficiency of the photodiode. The impact of such factors could be reduced by using a ratiometric detection scheme, in which signals from two different wavelengths or optical paths are measured simultaneously. The intensity ratio between the two channels could then be used to differentiate surface targets with different color contrasts while minimizing variations in reflected signal intensity caused by reflectivity and geometric changes. Addressing these factors will further improve detection reliability and treatment efficiency, advancing SPOT toward a more robust and broadly applicable platform for automated optical therapies.

SPOT is not limited to the applications demonstrated in this work. DR contrast represents just one class of optical signals that can be used to trigger treatment. In principle, this approach can be extended to other modalities, such as coherent Raman scattering, multiphoton fluorescence, or harmonic generation, which offer greater chemical specificity for target identification. Coupled with blue light or high‐energy pulsed lasers, these modalities could enable more selective, chemically targeted, and automated treatments.

The potential applications of SPOT also extend beyond skin treatments to areas such as interstitial laser surgery or PDT. In these contexts, SPOT could provide enhanced precision while minimizing unintended damage to surrounding healthy and vulnerable structures, such as nerves.

In cases where the target does not exhibit sufficient intrinsic DR or chemical contrast, externally applied markers, such as pigments or topical agents, can be used to define treatment regions. When combined with SPOT, this approach enables automated, spatially selective treatment with minimal risk of overtreatment.

In clinical use, SPOT can be integrated into existing laser‐based treatment systems by incorporating a guidance laser and a fast optical switch to control the treatment laser. It is worth noting that the intensity threshold may depend on treatment conditions and patient skin tone. We anticipate that, in advanced systems, standardized procedures will enable straightforward calibration and determination of these parameters.

## 5 Conclusion

In summary, we developed SPOT technology, a closed‐loop laser treatment system that uses DR detection to guide laser therapy in real time. By employing an intensity‐modulated guidance laser and lock‐in detection, SPOT enables sensitive detection of subtle optical contrasts while effectively rejecting ambient light interference, allowing real‐time decisions to be made during irradiation and minimizing potential collateral laser damage to healthy tissue areas. We demonstrated the capability of SPOT for automated pigment removal and targeted bacterial inhibition. These results highlight the ability of SPOT to achieve spatially selective and adaptive treatment based on highly sensitive surface contrast detection. This framework provides a generalizable strategy for integrating feedback control into laser therapies and may improve the safety, precision, and automation of laser use in a broad range of clinical laser therapy applications.

## Funding

This work was supported by the National Institute of General Medical Sciences (R35GM147092).

## Conflicts of Interest

Dr. Chi Zhang is the founder of Photokinesis LLC, a startup dedicated to commercializing advanced opto‐control technologies. The other authors declare no conflicts of interest.

## Supporting information


**Figure S1:** A dual‐channel comparator circuit box. The treatment laser activation voltage threshold can be fine‐tuned using the adjustment knob. The output of the comparator circuit can be inverted by the “invert” switch.
**Figure S2:** The darkness scale used in this study for ns laser treatment of laserjet printed patterns.
**Figure S3:** Optical configuration of the transient absorption (TA) microscope for imaging pigments. AOM, acousto‐optic modulator; PBS, polarization beam splitter; PD, photodiode.
**Figure S4:** Bright‐field transmission microscopy images and corresponding segmentation masks of *Pseudomonas aeruginosa* colonies from eight fields of view (FOVs 1–8) in untreated regions of the agar plate after overnight incubation.
**Figure S5:** Bright‐field transmission microscopy images and the corresponding segmentation masks from eight fields of view (FOVs 1–8) of *Pseudomonas aeruginosa* colonies grown in regions after being treated with a 405 nm continuous‐wave (CW) laser followed by overnight incubation.

**Video S1:** A video showing the selective removal of printed letters on paper with a background using SPOT.

## Data Availability

The data that support the findings of this study are available from the corresponding author upon reasonable request.
