# Supplementary material for: Automated Optical Treatment Based on Diffuse Reflectance Signals
Source: J Biophotonics. 2026 Sep 14;19(9):e70363. doi: 10.1002/jbio.70363 (PMC13574529; doi:10.1002/jbio.70363)
Supplement: Supplementary file 1 [file JBIO-19-e70363-s001.docx]

**Supporting Information**

**Automated optical treatment based on diffuse reflectance signals**

**Callum D. Appel^1^, Mark S. Carlsen^1^, Laura L. Lukov^1^, and Chi Zhang^1,2,3,*^**

*^1^James Tarpo Jr. and Margaret Tarpo Department of Chemistry, Purdue University, 560 Oval Dr., West Lafayette, IN 47907, USA*

*^2^Purdue Institute for Cancer Research, 201 S. University St., West Lafayette, IN 47907, USA.*

*^3^Purdue Institute of Inflammation, Immunology, and Infectious Disease, 207 S. Martin Jischke Dr., West Lafayette, IN 47907, USA.*

**zhan2017@purdue.edu*

**Sample Preparation**

For the paper-based samples used to evaluate SPOT system performance, a laserjet printer was employed to generate contrasts at varying darkness levels. In **Figure 2B**, the darkness percentage is created by changing ‘transparency’. For example, 100% darkness was achieved by creating a square in Microsoft Word and filling it with black color (hex code #000000) at 0% transparency, whereas 40% darkness corresponds to 60% transparency. For the automated removal of the “LASER” letters in **Figure 2C**, the letters were printed at 100% darkness on a 40% darkness background.

We observed that laserjet and inkjet printing produce distinctly different microscopic ink distributions. Inkjet printing tends to deposit more ink into deeper layers of the paper, whereas laserjet printing results in more surface-confined ink. However, due to the thinness of the paper substrate, the removal effects using a 1064 nm Nd:YAG nanosecond laser were similar for both printing methods. Therefore, only results from laserjet-printed samples are shown in the manuscript.

Synthetic skin (6”×8”, Autdor) was also used as a model system. Tattoos were created using a rotary tattoo machine (EK128B, Solong) with the provided black ink. The ink was deposited to an approximate depth of 0.5 mm into the synthetic skin, which has a total thickness of 1 mm. After tattooing, the surface was gently cleaned with deionized water and a paper towel to remove excess ink. Laser treatment was performed approximately 15 minutes after tattoo application.

Porcine skin samples were obtained from a local market and cut into approximately 2” × 3” sections. All samples were treated and imaged on the same day of purchase to minimize tissue degradation and moisture loss. Tattoos were applied at a depth of approximately 2 mm using the same rotary tattoo machine. After tattooing and before treatment, the tattooed porcine skin was cleaned with deionized water and a paper towel to remove excess ink on the surface. Treatment was conducted approximately 15 minutes after tattooing.

Bacterial samples were prepared by spreading a thin layer of *Pseudomonas aeruginosa* (MPAO1 strain, Manoil Lab mutant library) onto an approximately 0.8 mm thick agar layer. The dish bottom was marked with six small red dots to mimic skin redness in early-stage or mild erythema or other bacterial infections. Each dot is approximately 2 mm in diameter. Treatment was applied shortly after the bacterial layer dried, typically 15-20 minutes after deposition. The samples were then incubated overnight at 38 °C to facilitate colony growth and evaluate the inhibition effect. Images in **Figures 4C,D** were acquired after overnight incubation.

**Comparator Circuit**

Details of the comparator circuit configuration can be found in our previous publication [S1]. In this work, only one channel of a dual-channel comparator circuit was used. The input and output connections are illustrated in **Figure S1**.

Figure S1. A dual-channel comparator circuit box. The treatment laser activation voltage threshold can be fine-tuned using the adjustment knob. The output of the comparator circuit can be inverted by the ‘invert’ switch.

Figure S2. The darkness scale used in this study for ns laser treatment of laserjet printed patterns.

.

**Transient absorption microscopy:**

Figure S3. Optical configuration of the transient absorption (TA) microscope for imaging pigments. PBS, polarization beam splitter; AOM, acousto-optic modulator; PD, photodiode.

Transient absorption (TA) microscopy was performed to characterize ink particle morphology in samples before and after SPOT treatment. Due to their broad absorption spectrum, ink particles generate strong TA signals. The TA microscope was adapted from a stimulated Raman scattering (SRS) microscope in our lab [S2].

A femtosecond laser system (InSight X3+, Spectra-Physics) was used to provide both pump and probe beams, which were spatially combined before reaching the 2D galvo scanner (GVS002, Thorlabs). The pump and probe powers were set to 2 mW and 1 mW on the sample, respectively. An optical delay of approximately 30 ps was introduced between the pump and probe pulses to avoid SRS signals generated from the paper or synthetic skin samples. The pump beam was modulated at 2.5 MHz using an acousto-optic modulator (AOM, M1205-P80L-1 Isomet), and the resulting modulation transferred to the probe beam was detected using a digital lock-in amplifier (HF2LI, Zurich Instruments) at the same frequency. A short-pass filter (980 SP, Chroma Technology) was used to block the pump beam from reaching the photodiode. Images were acquired by raster scanning the combined lasers on the sample with ~2 s per frame and a pixel dwell time of 10 microseconds. For thicker synthetic skin samples, TA signals were collected using an epi-detection geometry, as shown in **Figure S3**.

Figure S4. Bright-field transmission microscopy images and corresponding segmentation masks of *Pseudomonas aeruginosa* colonies from eight fields of view (FOVs 1-8) in untreated regions of the agar plate after overnight incubation.

Figure S5. Bright-field transmission microscopy images and the corresponding segmentation masks from eight fields of view (FOVs 1-8) of *Pseudomonas* *aeruginosa* colonies grown in regions after being treated with a 405 nm continuous-wave (CW) laser followed by overnight incubation.

**Visualization 1 caption.**

**Visualization 1**. A video showing the selective removal of printed letters on paper with a background using SPOT.

**References**

[S1] B. Dong, S. Mahapatra, M.G. Clark, et al., and C. Zhang. "Spatiotemporal precise optical manipulation of intracellular molecular activities", *Advanced Science*, 2307342 (2024)

[S2] M.G. Clark, G. Gonzalez, and C. Zhang. "Pulse-picking multimodal nonlinear optical microscopy*". Analytical Chemistry* 94, 15405–15414 (2022).
